# Bystander Effect Induced by Electrical Stimulation Promotes N2a Differentiation Through Interleukin-6

**DOI:** 10.3390/bios16080447

**Published:** 2026-08-18

**Authors:** Daniel Martín, Luis Orta, Akaitz Dorronsoro, Diego Ruano, Alberto Yúfera, Paula Daza

**Affiliations:** 1 Departamento de Tecnología Electrónica, ETSII, Universidad de Sevilla, 41012 Sevilla, Spain; 2 Instituto de Microelectrónica de Sevilla, IMSE-CNM, CSIC/Universidad de Sevilla, 41092 Sevilla, Spain; 3 Departamento de Biología Celular, Facultad de Biología, Universidad de Sevilla, 41012 Sevilla, Spain; 4 Departamento de Bioquímica y Biología Molecular, Facultad de Farmacia, Universidad de Sevilla, 41012 Sevilla, Spain; 5 Instituto de Biomedicina de Sevilla (IBiS), Hospital Universitario Virgen del Rocío/Consejo Superior de Investigaciones Científicas/Universidad de Sevilla, 41013 Sevilla, Spain

**Keywords:** bystander effect, electrical stimulation, neuroblastoma, differentiation therapy

## Abstract

The bystander effect describes the induction of responses in non-targeted cells through cell signaling by directly stimulated cells. While this phenomenon has been extensively studied in the context of ionizing radiation, its occurrence following electrical stimulation (ES) remains poorly understood. Conditioned medium from N2a neuroblastoma cells exposed to voltage-controlled biphasic pulses at 500 mV/mm and 100 Hz induced neuronal differentiation in non-stimulated cells through an ES bystander effect. Bystander medium promoted morphological changes associated with neuronal differentiation, including increased neurite outgrowth and a reduction in the proliferation marker KI-67, indicating that the effects of ES extend to neighboring non-targeted cells. Molecular analysis revealed increased expression and secretion of interleukin-6 (IL-6) following ES, while neutralization of the IL-6 receptor inhibited the effects of ES, highlighting the role of IL-6 as a key mediator of this effect. We provide the first evidence that ES promotes a differentiation-associated bystander effect mediated by IL-6.

## 1. Introduction

The bystander effect is defined as the induction of biological changes in cells that have not been directly targeted by a specific stimulus but have received molecular signals from cells that were exposed. This phenomenon challenges the traditional “targeted” dogma of biology, which assumes that heritable effects and cellular damage require direct interaction with the initial stressor [1,2]. While the bystander effect is extensively documented in the context of ionizing radiation (IR) and ultraviolet (UV) light, its manifestation following electrical stimulation (ES) is a more recent and promising field of study [2].

In the well-established framework of radiation-induced bystander effects, irradiated cells release signaling molecules which can be transmitted: (1) via gap junctions, for molecules of up to 1000–1500 Da, which include ions such as Ca++, peptides, nucleotides, and other secondary messengers; and (2) through the release of soluble factors into the extracellular environment and their diffusion into the medium, including molecules like cytokines, such as IL-1, IL-6, IL-8, and IL-10, brain-derived neurotrophic factor (BDNF), tumor necrosis factor alpha, transforming growth factor, nitric oxide, and reactive oxygen species (ROS) [1,2,3]. All these signals trigger responses in unirradiated cells ranging from micronuclei, aberrations, and sister chromatid exchanges to apoptosis and cell cycle arrest [2,3]. Similar responses have been observed following UV irradiation, where oxidative stress and physical damage to the cell membrane prompt the release of mediators that affect unexposed cells in the same way [4]. Moreover, these consequences have also been observed in photodynamic therapy, heat, and chemotherapy agents; therefore, the bystander effect must be considered in a wide range of therapies to avoid the killing of non-treated cells [3].

Electrical stimulation (ES) is a technique that consists of applying an electric field (voltage, current, or charge) to living organisms, organs, tissues, or cell cultures. ES has been shown to promote specific cell responses, including adhesion, proliferation, migration, and differentiation [5,6]. On the other hand, ES is increasingly recognized not only as a tool for direct cellular manipulation but also as a potential inducer of a “bystander-like” effect.

ES-induced signaling is frequently associated with regenerative and therapeutic potential, offering a non-invasive pathway to enhance tissue repair [7], but the applications of ES are not limited to tissue engineering, and its capability to affect cell destiny could be very interesting in other fields like cancer differentiation therapy [8,9]. The advantage of this therapy lies in its ability to stop cancer progression, minimizing the harmful effects caused by traditional therapies (chemical or radiation), as it promotes cell differentiation without damage [10,11]. On the other hand, in the context of differentiation therapy, hormones or cytokines have been found to promote differentiation in vitro, modifying the phenotype of cancer cells [12]. In this way, it could be inferred that the study of the conditioned cell medium after ES could be of interest in trying to find new molecules that induce differentiation.

We have previously reported that neuroblastoma cells can be differentiated into neurons through ES; by using this methodology, we were able to induce the differentiation of N2a cells in both 2D and 3D, as well as halting proliferation without DNA damage [13]. Unlike the well-characterized radiation-induced bystander effect, which is generally associated with detrimental biological outcomes, the ES bystander effect under specific conditions of cellular differentiation, as previously described, may offer a therapeutic opportunity by suppressing cellular proliferation and thereby limiting cancer progression. This study focuses on the use of conditioned medium from electrically stimulated N2a cells in order to promote neuronal differentiation of non-stimulated N2a cells through a “bystander-like” effect and by trying to elucidate the main actor molecule of this effect.

## 2. Materials and Methods

### 2.1. Cell Culture

N2a (Neuro-2a) mouse neuroblastoma cells (ATCC CCL-131) were generously provided by Dr. Diego Ruano Caballero. The cells were routinely cultured in a 1:1 mixture of high-glucose Dulbecco’s Modified Eagle Medium (DMEM, Gibco 11965092, Grand Island, NY, USA) and Opti-MEM (Gibco 31985062), supplemented with 10% (*v*/*v*) fetal bovine serum (FBS, Gibco A5256701), 2mM L-glutamine (Gibco 25030081), 50 μg/mL streptomycin (Gibco 15140122), and 50 U/mL penicillin (Gibco 15140122). Cultures were maintained at 37 °C in a humidified incubator with 5% CO_2_. The cells were routinely passaged to ensure cells were in the exponential growth phase when used for experiments.

**Figure 1:**
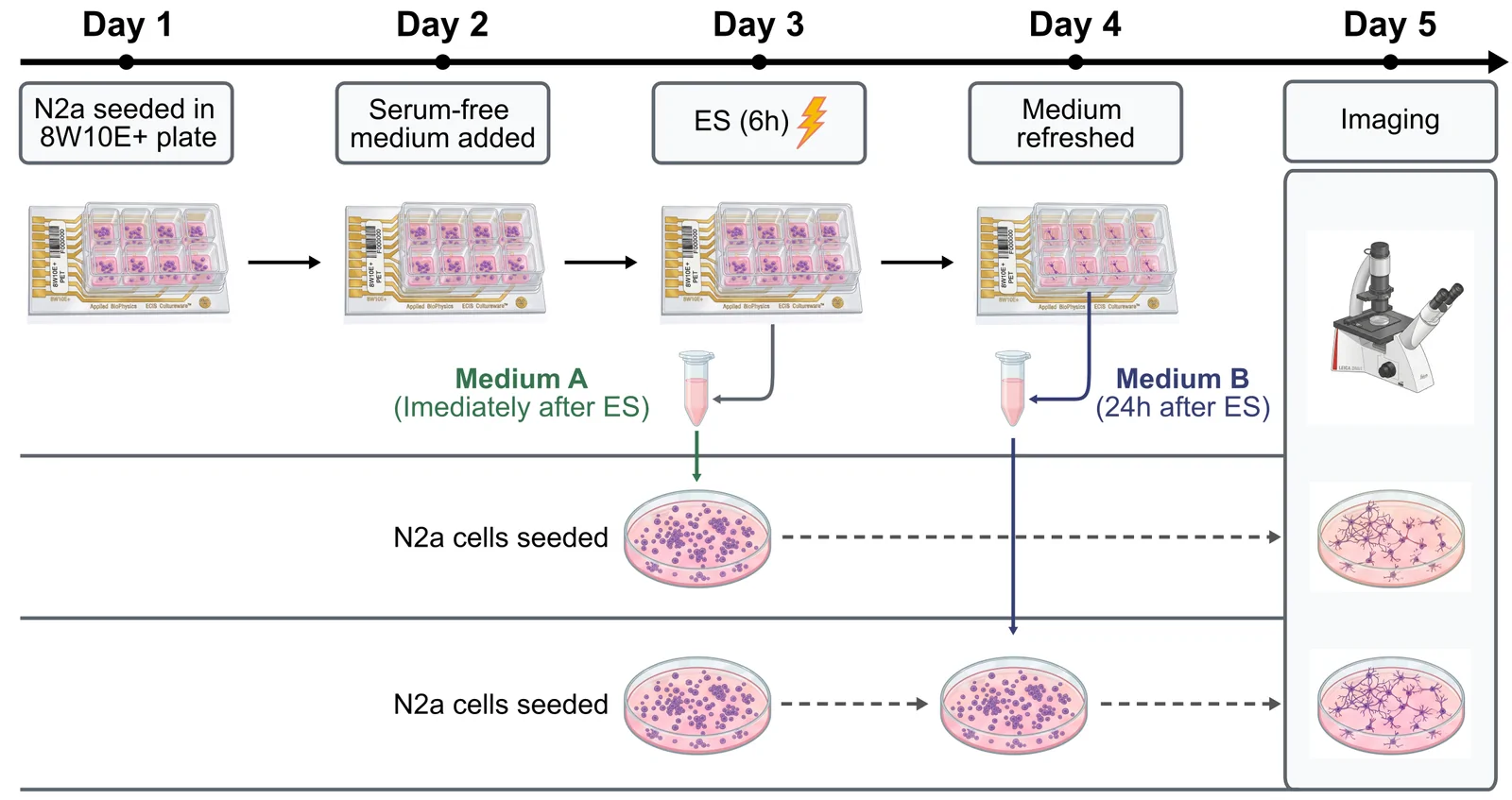
Bystander experimental protocol.

10,000 N2a cells were seeded in each well of an 8W10E+ plate. The following day, the medium was replaced with serum-free medium. On the third day, the cells were exposed to ES for 6 h, and 75,000 cells were seeded in separate 35 mm Petri dishes. Immediately after the 6 h ES treatment, the medium from the 8W10E+ plate (medium A) was transferred to the corresponding seeded dishes for both the control and ES conditions. The medium in the 8W10E+ plate was then refreshed. Approximately 24 h after ES, the medium from the electrode plate was transferred to two additional Petri dishes (medium B). Images of the cells in the Petri dishes were taken on day five. The images were analyzed using ImageJ (version 1.54f) software. A diagram of the experimental protocol can be seen in Figure 1.

Recombinant mouse IL-6 (R&D, 406-ML/CF) was added at a concentration of 50 ng/mL 2 h prior to ES to the conditions that required it. For the inhibition of the IL-6 receptor, 1 h prior to ES, the cells were treated with 1 µg/mL of IL-6 receptor blocking antibody (R&D, AF1830). This blocking antibody will be referred to as αIL-6R.

### 2.2. Electrical Stimulation

Electrical stimulation was performed as described in [13]. Briefly, the applied ES signals were biphasic square voltage signals with a duty cycle of 50% (25% positive and 25% negative) at 100 Hz, with an amplitude of ±500 mV. These signals were previously studied by the authors in [6], where many amplitudes and frequencies were tested and characterized; 500 mV–100 Hz was found to be the best condition for neuronal differentiation of N2a cells without producing DNA damage.

### 2.3. Morphological Analysis Method

For morphological analysis, images were taken with a 20× objective in an inverted microscope (Leica, Wetzlar, Germany). In order to obtain the cell differentiation percentage, differentiated and nondifferentiated cells on top of the electrode were counted using ImageJ software, as described in [5]. The differentiation percentage was calculated as the number of cells displaying a differentiated morphology divided by the total number of cells counted. Neurite length was measured using the ruler tool in ImageJ and expressed in µm.

### 2.4. Cytometric Bead Array Assay

Cytokine levels were quantified using the BD™ Cytometric Bead Array (CBA) Mouse Inflammation Kit (BD Biosciences, San Jose, CA, USA, Cat. No. 552364), according to the manufacturer’s instructions. Briefly, 10 µL of the bystander medium samples and cytokine standards were incubated with a mixture of capture beads coated with cytokine-specific antibodies, followed by incubation with phycoerythrin (PE) conjugated detection antibodies. After washing, the samples were analyzed by flow cytometry (Miltenyi MACSQuant VYB). Cytokine concentrations were determined by comparison to the standard curves generated.

### 2.5. RT-qPCR

The cells were lysed using Trizol reagent (gTPzol01 CViral). RNA was isolated 24 h after ES started, using the E.Z.N.A. Total RNA Kit I (Omega Bio-Tek, Norcross, GA, USA). Total RNA was reverse transcribed with the iScript cDNA Synthesis Kit (Bio-Rad, Hecules, CA, USA). The concentration of the extracted RNA was determined using a NanoDrop spectrophotometer. The real-time PCR amplification was done in a CFX Connect Real-Time System (Bio-Rad) using SYBRGreen (Bio-Rad), and the cDNA was obtained as a template. Primer sequences can be found in the Supplementary Information.

### 2.6. Immunofluorescence

The cells were washed with PBS and fixed for 30 min with 4% paraformaldehyde in PBS, then permeabilized for 10 min with 0.5% Triton-X. After this, the cells were treated with the blocking solution (1% bovine serum albumin + 0.1% Tween 20 in PBS) for 15 min. The primary antibody for KI-67 (BD Pharmingen 550609, San Diego, CA, USA) was diluted 1:500 in the blocking solution. The cells were incubated with this antibody at 4 °C overnight, after which they were washed 3 times with the blocking solution. The same process was followed for the secondary antibodies (Invitrogen A32731, Waltham, MA, USA), diluted 1:500 in the blocking solution, and incubated for 45 min. DAPI staining was carried out after the secondary antibody incubation. Immunofluorescence (IF) images were taken with a Leica DMi8 microscope with a 63× objective. Images were analyzed with Cell Profiler, where the nuclei were isolated using the DAPI channel and green fluorescence was measured inside the nucleus.

### 2.7. Statistical Analysis

The data are represented as boxplots or raincloud plots; the median Q1 and Q3 are represented by solid or dashed lines, respectively. Each experiment was independently performed at least in duplicate. All statistical analyses were performed using a one-way analysis of variance (ANOVA) test, where *p*-values < 0.05 were considered statistically significant. Unless otherwise noted, all tests have been performed with reference to the control condition.

## 3. Results

### 3.1. ES Can Provoke a Bystander Effect

**Figure 2:**
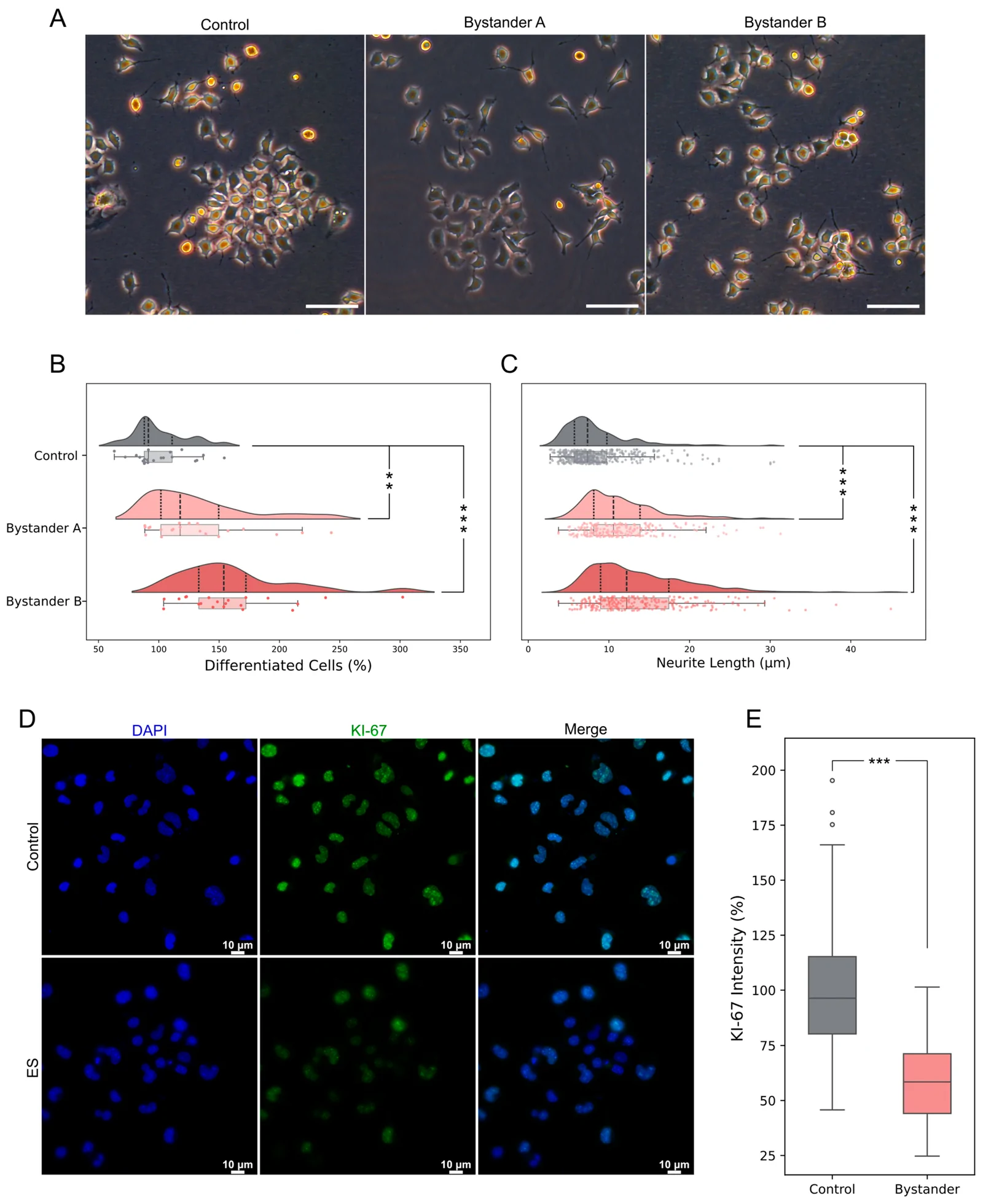
The bystander effect morphological assessment and KI-67 analysis. (**A**) Phase contrast microscopy images taken at 20X with a 2X digital zoom. The scale bar represents 50 μm. Quantitative analysis of the differentiated cells (**B**) (** *p* < 0.01, *** *p* < 0.001; *n* = 21) and neurite length (**C**) (*** *p* < 0.001; *n* = 200). (**D**) Representative immunofluorescence images of KI-67 (green) and DAPI (blue), images taken at 63X, and the scale bar represents 20 µm. (**E**) Quantification of KI-67 intensity represented as a percentage of the control (*** *p* < 0.001; *n* = 63).

Firstly, the ES-induced bystander effect was characterized. For this, the medium from electrically stimulated cells was transferred to non-stimulated cells, right after ES (bystander medium A) and 24 h after the start of ES (bystander medium B). The separation of medium A and B allows us to distinguish between the effects produced by electrochemical byproducts, if present (medium A), and mostly cell secretion (medium B). At 24 h after the last medium transfer, images of bystander cells were taken and analyzed. Representative images are displayed in Figure 2A. The control cells were tightly packed and exhibited occasional neurites, while largely retaining a mostly undifferentiated morphology. On the contrary, the bystander cells displayed a more polygonal shape, with more frequent and longer neurites. Quantification of cells displaying differentiated morphology and neurite length can be seen in Figure 2B,C. Both bystander media induced the differentiation and neurite outgrowth of N2a cells, although medium B was more effective, with 150% differentiated cells compared to the control. The cells exposed to medium B exhibited an increase in differentiation percentage of around 50% compared to the control, as well as a significant increase in neurite length. Next, alterations in proliferative capabilities produced by the bystander effect were characterized by KI-67 immunofluorescence. Bystander medium was transferred 24 h after ES, as medium B was the most effective. KI-67 IF representative images can be seen in Figure 2D. The intensity of KI-67 was significantly lower in the bystander cells (Figure 2E). The bystander cells displayed a decrease in KI-67 IF intensity of around 60% compared to the control. This is consistent with the morphological analysis, as neuronal differentiation entails the arrest of the cell cycle and thus a decrease in KI-67.

### 3.2. ES Bystander Effect May Be Mediated by Interleukins

**Figure 3:**
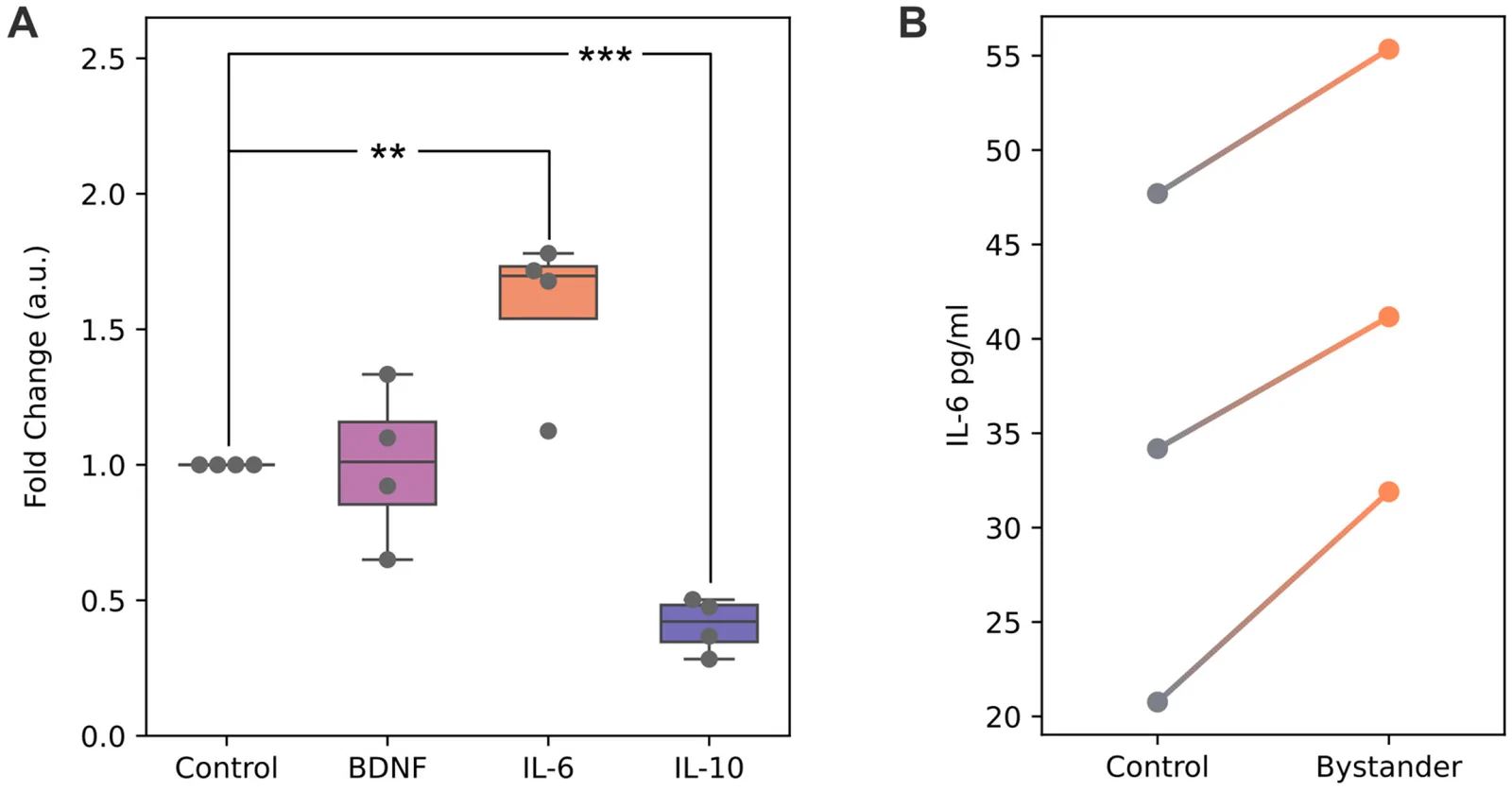
Molecular analysis of cell excretion and bystander medium. (**A**) RT-qPCR of interleukins and BDNF in directly stimulated cells (** *p* < 0.01, *** *p* < 0.001; *n* = 4). (**B**) Cytometric Bead Array analysis of IL-6 concentration in bystander medium; paired experiments are represented with lines.

After analyzing the effect of the bystander effect, the content of this medium was assessed. RT-qPCR analysis of BDNF, IL-6, and IL-10 was performed in the ES and control cells (Figure 3A). No change was observed in BDNF expression. IL-6 was upregulated in the ES cells, with a consequent reduction in IL-10 expression. Furthermore, IL-6 concentration in the bystander medium was assessed by Cytometric Bead Array analysis (Figure 3B) and found to be higher than in the control. Although the absolute value of the concentration was different in each replicate, it was always higher in the bystander medium. These results point to IL-6 as a possible candidate mediating the bystander effect, as the ES cells produced and secreted it.

### 3.3. Interleukin-6 Is the Main Cytokine Implicated in the ES Bystander Effect

**Figure 4:**
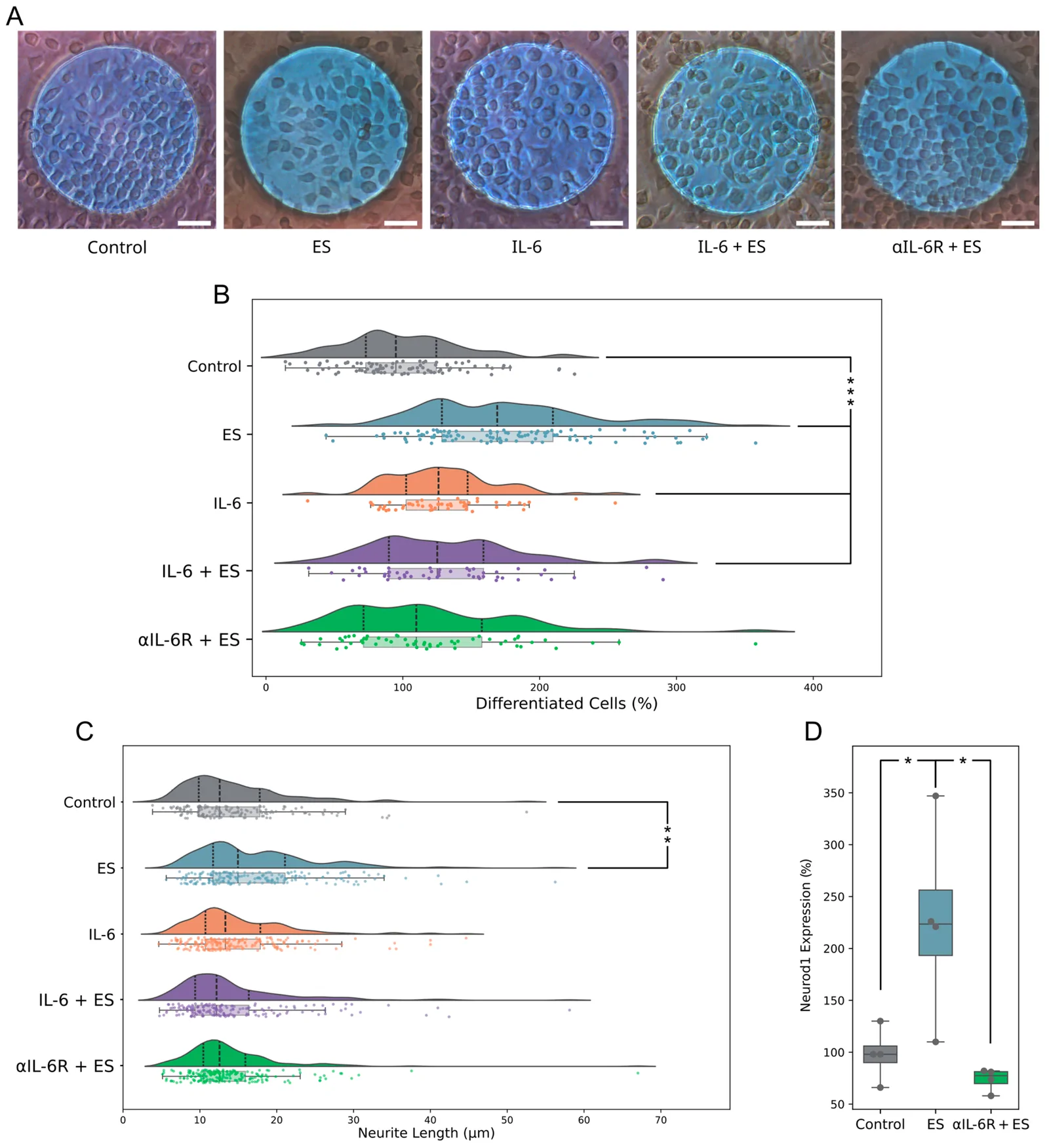
Effect of recombinant IL-6 and neutralization of IL-6R in combination with ES. (**A**) Phase contrast microscopy representative images. Scale bar represents 50 µm. Quantitative analysis of differentiated cells (**B**) (*** *p* < 0.001; *n* = 60) and neurite length (**C**) (** *p* < 0.01; *n* = 151). (**D**) RT-qPCR analysis of Neurod1 expression (* *p* < 0.05; *n* = 4).

To confirm the role of IL-6 in the bystander effect produced by ES, combinatory treatments of ES with 50 ng/mL of recombinant IL-6 and IL-6 receptor blocking antibodies were tested. As recombinant IL-6 does not have the same biological activity as endogenous IL-6, the dose chosen had to be significantly higher than the one observed in the conditioned medium. Representative images are displayed in Figure 4A. Again, the control cells were tightly packed and exhibited occasional neurites with mainly undifferentiated morphology. On the contrary, the ES cells displayed a more polygonal shape with more and longer neurites. The cells treated with 50 ng/mL of recombinant IL-6 exhibited an intermediate morphology between the control and ES-treated cells, but a significant difference with respect to control cell morphology was observed. While a variety of cells displayed a differentiated phenotype, some retained a morphology similar to the control cells and, overall, neurites were not as long, regardless of whether IL-6 was administered alone or in combination with ES. Lastly, stimulated cells in the presence of the αIL-6R remained very similar to the control, with few neurite processes. This is confirmed by the morphological analysis in Figure 4B. ES induced the most significant difference in morphologically differentiated cells, with a 160% increase compared to the control. Recombinant IL-6 produced an increase in differentiation regardless of its combination with ES, which was slightly lower than the ES cells but also statistically significant. αIL-6R suppressed the effects of ES on N2a morphology. A very similar outcome can be observed when analyzing neurite length (Figure 4C). In this case, the only significant change regarding the control was observed for the ES condition. Recombinant IL-6 alone also produced a very slight increase in neurite length. Lastly, αIL-6R suppressed the effects of ES on neurite length.

The effect of the αIL-6R was further confirmed by Neurod1 RT-qPCR. Neurod1 is a neuronal differentiation onset marker that was overexpressed in ES cells and suppressed by the αIL-6R (Figure 4D). These results clearly indicate the involvement of IL-6 in the induction of neuronal differentiation produced by the ES bystander effect.

## 4. Discussion

The ES-induced bystander effect was first evaluated through morphological analysis and was found to provoke an increase in differentiated cells. KI-67 immunofluorescence confirmed that the ES bystander medium produced a decrease in proliferating cells previously associated with neuronal differentiation [13]. We have previously shown that ES reduced the proliferative capabilities of N2a cells both in monolayers and in neurospheres. This effect could be linked to the neuronal differentiation of these neuroblastoma cells, since, as progenitor cells progress through the differentiation process, their proliferation capabilities are reduced [13]. These new results relating to differentiation therapy expand the traditional understanding of bystander signaling into the field of electrical stimulation, which, as we have previously described, has been extensively documented in response to ionizing radiation and UV light. They indicate that ES acts not only on the directly stimulated cells but also on the surrounding population via cellular communication. The distinction between the bystander media extracted right after ES and the next day provided insight into the nature of the signaling mechanism. While both media induced some level of differentiation, the medium containing mostly cell secretions appeared more effective, suggesting that the primary driver of differentiation is likely a biological response involving cell-secreted factors rather than transient electrochemical byproducts generated during ES delivery. This is consistent with other research indicating that while ES can generate ROS in the culture medium, these factors alone may be insufficient to fully replicate the effects observed with direct stimulation [14].

The use of ES-conditioned medium as a catalyst for the bystander effect is a novel approach, but precedents exist. Research indicates that ES can significantly enhance the secretion of extracellular vesicles and different molecules that modulate the physiology of distant cells [4,15]. For example, conditioned medium derived from ES-treated mesenchymal stem cells has demonstrated the ability to inhibit tumor cell migration and 3D spheroid growth, and promote bone healing in unexposed tissues [16]. Furthermore, in cardiac models, electrical stimulation increases the release of cardioprotective EVs that shield non-stimulated cardiomyocytes from hypoxia-induced apoptosis [17]. In [18], ES-conditioned medium from Schwann cells promoted sustained neurite outgrowth in a neuronal culture. Similarly, conditioned medium obtained from electrically stimulated mesenchymal stem cells and Jurkat T cells showed tumor-suppressive activity, reducing tumor cell migration, 3D spheroid growth, and cancer tissue fragment viability [16]. In both studies, medium from ES cells is transferred to a different cell line. In this study, the same cell line is used to generate the bystander medium and to assess its effect on it, in an attempt to standardize a possible protocol to halt cell progression in neuroblastoma cells.

Molecular analysis pointed toward IL-6 as a possible contributor to this ES-induced bystander effect. While IL-6 is frequently characterized as a proinflammatory cytokine in various bystander models, it is known to function as a neurotrophin molecule within the central nervous system [19]. Higher concentrations of IL-6 were found in the ES bystander medium as per the Cytometric Bead Array assay. This correlates with [20], where a higher concentration of IL-6 was found in ES mesenchymal stem cells via cytokine arrays. Recombinant IL-6 was also responsible for a slight increase in N2a differentiation, consistent with [21,22], where IL-6 was shown to induce the neuronal differentiation of PC12 and SH-SY5Y cells, respectively. The observed upregulation of IL-6 in the ES-treated cells and its increased concentration in the bystander medium suggest a similar role in N2a cells, potentially triggering the transition toward a mature phenotype. This was further confirmed by the inhibition of the IL-6 receptor using a blocking antibody. This inhibitor cancelled the induction of differentiation produced by ES as detected by morphological analysis and Neurod1 RT-qPCR.

No earlier evidence pointing to Il-6 as a contributor to the ES-induced bystander effect has been found. Other authors have explained the effect of the ES bystander medium through the Piezo1 pathway [16]. This pathway has been associated with ES in several studies, as Piezo1 is a protein that reacts to physical stimuli [14,16]. Furthermore, the Piezo1 pathway and IL-6 have been related. Specifically, recent research in skeletal muscle models indicates that ES mimics the effects of mechanical exercise and induces the secretion of IL-6 in a Piezo1-dependent manner [23]. The connection between Piezo1 and IL-6 pathways has been attributed to calcium ions [24], which are also involved in the neuronal differentiation induction produced by ES [25]. This evidence points to IL-6 as possibly responsible for the ES bystander effect produced in N2a, probably through a Piezo1-dependent mechanism.

More studies must be performed to determine the exact role of IL-6 and possibly Piezo1 as differentiating agents. On the other hand, as we have previously described, extracellular vesicles generated by ES could play a relevant role in the modulation of the physiology of distant cells [15], and it would be of great interest to assess the possible presence of IL6 in exosomes secreted by the cells after ES. Furthermore, in our opinion, it would be very useful to try to establish a role for this described bystander effect as a possible therapy for cancer progression.

## 5. Conclusions

This study focused on characterizing the ES-induced bystander effect in N2a cells. It was shown that the ES bystander effect could promote neuronal differentiation in neuroblastoma cells through soluble factors released by directly stimulated cells. Bystander medium from ES-treated N2a cells induced morphological changes consistent with neuronal differentiation, including increased neurite outgrowth and a reduction in the proliferation marker KI-67, indicating that ES-mediated signaling extends beyond the directly stimulated cell population. Molecular analysis identified IL-6 as a possible contributor to this response, as ES increased both its expression and secretion, while neutralization of the IL-6 receptor inhibited the effects of ES on neuronal differentiation. These findings provide the first evidence that IL-6 participates in an ES-induced bystander effect associated with neuronal differentiation. From a therapeutic perspective, the ability of ES to trigger differentiation indirectly through paracrine signaling may broaden its potential application as a non-genotoxic agent for differentiation therapy. Nevertheless, this study was performed in a single in vitro cell line and focused primarily on soluble cytokine signaling. Further studies are required to validate these findings in additional models, determine the contribution of extracellular vesicles and other signaling pathways, and assess the relevance of this mechanism in vivo.

## Data Availability

The datasets used and/or analyzed during the current study are available from the corresponding author on reasonable request.

## Supplementary Materials

The following supporting information can be downloaded at: https://www.mdpi.com/article/10.3390/bios16080447/s1.
