# Supplementary material for: Bystander Effect Induced by Electrical Stimulation Promotes N2a Differentiation Through Interleukin-6
Source: Biosensors (Basel). 2026 Aug 18;16(8):447. doi: 10.3390/bios16080447 (PMC13511692; doi:10.3390/bios16080447)
Supplement: Supplementary file 1 [file biosensors-16-00447-s001.zip › biosensors-4475077-supplementary.pdf]

RT-qPCR Primers

Gene	Forward	Reverse
Neurod1	TCAGCATCAATGGCAACTTC	AAGATTGATCCGTGGCTTTG
IL-6	CTGGGGATGTCTGTAGCTCA	CTGTGAAGTCTGCTGTCCGG
IL-10	ACCTGGTAGAAGTGATGCCC	ACACCTTGGTCTTGGAGCTT
mRplp0	CCAGGCTTTGGGCATCAC	CTCGCTGGCTCCCACCTT
